# *Lactobacillus rhamnosus* GG Alleviates Colitis by SLC5A12-Mediated Th17/Treg Cell Balance in Mice

**DOI:** 10.3390/nu18111724

**Published:** 2026-05-28

**Authors:** Yiling Zhang, Xianghong He, Qian Zhao, Qiming Duan, Heping Li, Rui Qin, Weifang Zuo, Kunhong Xie, Bo Han

**Affiliations:** 1 School of Pharmacy, Chengdu University of Traditional Chinese Medicine, Chengdu 611137, China; zhang.yiling2023@stu.cdutcm.edu.cn (Y.Z.); hexianghong@cdutcm.edu.cn (X.H.); zhaoqian@cdutcm.edu.cn (Q.Z.); liheping0210@foxmail.com (H.L.); qinrui831@163.com (R.Q.); zuoweifang@cdutcm.edu.cn (W.Z.); 2 CDUTCM-KEELE Joint Health and Medical Sciences Institute, Chengdu 611137, China; 3 Key Laboratory of Animal Nutrition and Efficient Utilization of Feed Nutrients, Chengdu 625014, China; qimingduan0910@163.com

**Keywords:** LGG, colitis, Th17 cells, Treg cells, SLC5A12

## Abstract

**Background/Objectives**: *Lactobacillus rhamnosus* GG (LGG) is one of the most widely utilized probiotic strains with a variety of biological functions including prevention and treatment of gastro-intestinal infections and regulation of immune responses. **Methods**: Here, we explored the role of LGG in regulating the differentiation of naïve CD4^+^ T cells and its effect on alleviating the dextran sulfate sodium (DSS)-induced colitis in mice. **Results**: In vitro, we showed that LGG-derived metabolites not only promoted the differentiation of naive CD4^+^ T cells into T-helper 17 cells (Th17 cells), but also selectively upregulated the expression of lactate-specific transporter solute carrier family 5 member 12 (SLC5A12). Interestingly, we manipulated a CD4^+^ T cell-monocytes co-culture and found that heated LGG-loaded monocytes modulate naive CD4^+^ T cells to differentiate preferentially into Treg cells rather than Th17 cells. To explain the above-mentioned contradiction, we used an experimental colitis model and found that LGG administration alleviated the DSS-induced colitis in mice, as indicated by decreases in weight loss and disease activity index. Moreover, SLC5A12 blockade (using a specific antibody) further reduced the colonic histological inflammatory score and decreased secretion of proinflammatory cytokines such as IFN-γ, IL-6, IL-17F, and IL-21. Notably, SLC5A12 blockade abolished the LGG-induced differentiation of the IL-17^+^CD4^+^ T (Th17) cells but significantly increased the frequency of Foxp3^+^CD4^+^ T (Treg) cells in the colonic lamina propria. Furthermore, a higher intracellular lactate concentration was observed in the colonic CD4^+^ T cells isolated from the LGG-treated colitic mice compared with other groups. Additionally, we also found elevated levels of oxidative stress indicators such as MDA and H_2_O_2_, as well as excessive reactive oxygen species (ROS) in colonic tissue of DSS-treated only mice, while LGG can scavenge ROS by inducing nuclear factor-erythroid 2-related factor 2 (Nrf2) expression in enterocytes. **Conclusions**: Altogether, these results indicate that LGG might alleviate preclinical colitis by modulating the Th17/Treg balance, and SLC5A12 blockade appears to enhance the anti-inflammatory properties of LGG.

## 1. Introduction

The pathology of idiopathic inflammatory bowel disorders (IBD), such as ulcerative colitis (UC), involves immune cell activation and disturbances in cytokine production secondary to impaired barrier function in the intestinal mucosa. During the development of UC, T cells serve as a major source of cytokines, leading to an imbalance between pro-inflammatory cytokines, such as IL-17A produced by Th17 cells, and anti-inflammatory cytokines, such as IL-10 produced by Treg cells [1]. Atarashi et al. discovered that enterohemorrhagic *Escherichia coli* (EHEC) O157:H7, isolated from patients with UC, triggers a gene expression program in the epithelium that induces a Th17 cell response [2]. Fortunately, such abnormal mucosal immune responses are usually suppressed by peripheral Treg cells, which contribute to the secretion of the anti-inflammatory cytokines such as TGF-β and IL-10 and thus functionally support intestinal immune homeostasis [3]. In clinical applications, the therapeutic potential of probiotics for UC has received considerable attention [4,5]. In vivo murine trials have shown that supplementation with probiotics can promote gut health by multiple means, including protection against pathogens, maintenance of epithelium barrier function, and improvement of immune cell balance [4]. Nevertheless, an increasing body of research also reports that the effectiveness of probiotics is often limited by insufficient colonization at the inflammatory site, and this low colonization, in turn, is further exacerbated by the excessive generation of oxygen-free radicals [6].

Among the different commercially available bacterial strains observed, *Lactobacillus rhamnosus* GG (LGG) is the preferred strain for probiotic products, and part of the reason lies in its potent antioxidant properties, resistance to gastric acid, and excellent colonization ability on intestinal epithelial villi [7]. Its various health effects, including the prevention and attenuation of various inflammatory diseases such as allergies [8], pneumonia [9], and diarrhea [10], have been extensively highlighted. More strikingly, LGG is already a clinically used probiotic with prior evidence of efficacy in antibiotic-associated diarrhea [10], *Helicobacter pylori*-related dyspepsia [11], infant colic [12], and other gastrointestinal indications [13]. Concurrently, studies have postulated that LGG could exert anti-inflammatory effects through diverse mechanisms, especially by regulating the host immune response [14]. As a matter of fact, LGG could reduce the neutrophil infiltration triggered by CD4 activation via modulating the adaptive immune response in mice [15]. Encouragingly, probiotic LGG-CICC 6141 has been reported to downregulate the Th17/Treg ratio in colonic lamina propria (cLP) via the Toll-like receptor 2 (TLR2) pathway, thereby mitigating DSS-induced colitis in mice [16]. These results position LGG as a potential orchestrator of intestinal Th17/Treg homeostasis in the lamina propria.

Over the past few years, the relationship between the Th17/Treg balance and certain bacterial strains has become increasingly clear, as evidenced by the ongoing accumulation of evidence. For example, the complex, defined, community-induced Treg cells and Th17 cells with epitope specificity were screened through every strain T cell receptor (TCR) from constructed T cell hybridomas, as reported by Nagashima et al. (2023) [17]. According to their research, *Lactococcus lactis* subsp. *lactis* II1403-specific pTh17 cells were observed in the intestinal T cell pool of germ-free mice colonized with defined bacterial strains. Likewise, in a newborn mouse model, IL17-committed γδ T cells elicited in the lung have an invariant Vγ6Vδ1 TCR specific for an epitope from the LGG [18]. By contrast, LGG is reported to indirectly induce a Treg cell response; LGG expands some taxa of intestinal short-chain fatty acids (SCFAs)-producing bacteria, which selectively augment the Treg response [19]. Intriguingly, LGG generates millimolar concentrations of lactate, a key metabolic product, and several studies emphasize lactate’s role in initiating Th17 cell differentiation [20]. The transport of lactate is mediated by six known solute carrier transporters, including Monocarboxylate transporter 1–4 (MCT1–4) or Solute Carrier Family 5 Member 8 and 12 (SLC5A8, SLC5A12). Notably, Pucino et al. described a mechanism by which lactate initiates Th17 cell differentiation, where the sodium-dependent transporter SLC5A12—which transports lactate across the plasma membrane—activates Th17 cell proliferation [21]. Although SLC5A12 is typically highly expressed in the kidney, retina, and colon, recent research indicates that under inflammatory conditions such as arthritis and aging, its expression is enhanced on the membrane of various CD4^+^ T helper cells, including Th1 and Th17 cells [22,23]. This finding raises the hypothesis that LGG may be of great significance for the therapy of preclinical colitis characterized by a Th17/Treg imbalance, which is closely related to the lactate transporter SLC5A12. Thus, this study, which decodes the precise immunoregulatory mechanisms of LGG in the context of DSS-induced colitis, could address this hypothesis while providing a new theoretical basis for applying probiotic therapy for preclinical colitis.

## 2. Materials and Methods

### 2.1. Mice

Eight-week-old male mice, purchased from Dashuo Experimental Animal Co., Ltd. (Chengdu, China), were on the C57Bl/6 background. Mice were maintained in our facility under specific-pathogen-free (SPF) conditions on a 12-h dark/light cycle schedule in a temperature-controlled (23–25 °C) and humidity-controlled room. In addition, mice had access to a sterilized laboratory rodent diet and autoclaved distilled water ad libitum. The animal experimental procedures and protocols were approved by the Animal Care and Use Committee of the Sichuan Agricultural University and were conducted in accordance with the Guidelines for the Care and Use of Laboratory Animals.

### 2.2. Bacterial Strain and Culture

LGG (ATCC 14917), purchased from American Type Culture Collection (ATCC; Manassas, VA, USA), was anaerobically cultured in de Man, Rogosa, and Sharpe (MRS) medium as appropriate in a shaking incubator at 37 °C. Quantitative analysis of LGG in MRS broth was performed by a 0.5 A600 reading of cultures at known concentrations, which were tested by serial dilutions and plating for colony-forming unit counts on MRS agar. For preparation of bacterial supernatant, LGG was cultured to reach log-phase growth, then centrifuged at 12,000× rpm for 10 min, and subsequently, the culture supernatant used for the CD4^+^ T cell culture assay was obtained through a 0.22 μm filter. For murine gavage, LGG under logarithmic growth phase was collected and centrifuged at 3000× *g* for 10 min. After the supernatant was aspirated, the bacterial pellet was washed three times with cold PBS, then re-suspended with PBS to reach the desired number of LGG (1 × 10^9^ CFU) per 100 μL.

### 2.3. Primary T Cells Culture and Treatment

For isolation of naive CD4^+^ T cells (CD45^+^ CD4^+^ CD44_low_ CD62L_high_), cells were collected from spleens and lymph nodes of mice, and then stained with antibody solutions including anti-CD45 (clone 30-F11), anti-CD4 (clone GK1.5), anti-CD44 (clone IM7), and anti-CD62L (clone MEL-14) for 40 min at 4 °C in the dark. After incubation, these stained cells were sorted on a BD FACSAria III cell sorter (BD Biosciences, Franklin Lakes, NJ, USA), and the purity of the sorted naive CD4^+^ T cells was >90%. The isolated naive CD4^+^ T cells were cultured in RPMI media supplemented with 10% FBS (Gibco, Grand Island, NY, USA), 25 mM glutamine (Agilent, Santa Clara, CA, USA), 500 units of penicillin–streptomycin (Gibco, Grand Island, NY, USA), and 50 μM β-mercaptoethanol (Sigama, Darmstadt, Germany). Before T cell polarization, naive CD4^+^ T cells were seeded at 100,000 cells/well in 24-well plates and activated for 2 days with plate-bound anti-CD3 (0.2 μg/mL; (BioLegend, San Diego, CA, USA)) and soluble 1.5 μg/mL anti-CD28 (BD Biosciences, Franklin Lakes, NJ, USA). For Th1 cell generation, naive CD4^+^ T cells were cultured for the following 3 days in standard RPMI media in the presence of 20 ng/mL recombinant mouse IL-2 (BioLegend, San Diego, CA, USA), and 5 μg/mL anti-IL-4 (BioLegend, San Diego, CA, USA). For Th2 cell polarization, naive CD4^+^ T cells were cultured for the following 3 days in standard RPMI media in the presence of 5 μg/mL rmIL-2, 50 ng/mL recombinant mouse IL-4 (BioLegend, San Diego, CA, USA) and 5 μg/mL anti-IFN-γ (BioLegend, San Diego, CA, USA); Treg cells were polarized by culturing naive CD4^+^ T cells for 3 days in standard RPMI media with 200 U/mL recombinant human IL-2 (BioLegend) and 1 ng/mL recombinant human TGF-β1 (BioLegend, San Diego, CA, USA). For Th17 cell induction, naive CD4^+^ T cells were cultured for the following 3 days in standard RPMI media containing 10 μg/mL recombinant mouse IL-6 (BioLegend, San Diego, CA, USA), 1 ng/mL rhTGF-β1, and 5 μg/mL anti-IFN-γ and 5 μg/mL anti-IL-4. During the polarization experiment, naive CD4^+^ T cells were differentiated in the presence of lactate (1 mM, 5 mM, 10 mM) or bacterial culture supernatants, which were added together with standardized cytokine combinations. Specifically, 50 μL of LGG supernatant was added to 500 μL of cell culture.

### 2.4. Experimental DSS-Induced Colitis and Study Design

For the acute murine colitis model, C57BL/6J mice were administered 2.5% (*w*/*v*) DSS (MP Biomedicals, Santa Ana, CA, USA) in sterile drinking water for 8 days to induce inflammation. Forty mice were randomly divided into four groups (*n* = 10), Con group: mice were intragastrically administered 100 μL MRS media daily and treated by intraperitoneal injection of 20 μL of SLC5A12’s isotype antibody (0.1 mg/mL, Aibersen, Beijing, China) on day 3; DSS group: mice were intragastrically administered 100 μL MRS media from one week before the DSS treatment to the day before the end of the experiment, and treated by intraperitoneal injection of 20 μL of SLC5A12’s isotype antibody on the third day post-DSS treatment; LGG + Iso-SLC5A12 Ab group: mice were intragastrically administered 100 μL MRS media containing live LGG (10^9^ CFU) from one week before the DSS induction to the day before the end of the experiment, and treated by intraperitoneal injection of 20 μL of SLC5A12’s isotype antibody on the third day post-DSS treatment; LGG + SLC5A12 Ab group: mice were intragastrically administered 100 μL MRS media containing live LGG from one week before the DSS induction to the day before the end of the experiment, and treated by intraperitoneal injection of 20 μL of anti-SLC5A12 (0.1 mg/mL, clone 7C1 (Aibersen, Beijing, China)) on the third day post-DSS induction. During the DSS treatment, weight loss was monitored, and the feces of each mouse were collected to observe consistency and the presence of blood in the stool daily. The disease activity index (DAI) of each mouse was assessed daily according to the method described before [24]. At the end of the experiment, mice were euthanized, and their colons were excised and collected for histopathological, cytokine, and immunological flow cytometry (FCM) analyses.

### 2.5. Histopathological Analysis

For histologic analysis, the initial step was the fixation of colon samples with a 4% paraformaldehyde solution at room temperature for 24 h. Following this, the tissues underwent dehydration in graded alcohol solutions and were then embedded in paraffin. After being cut into sections of 3–5 μm using a microtome, these ultrathin slices were stained with hematoxylin and eosin (H&E) and sealed with eosin. The stained slices were finally photographed as photomicrographs using a pathologic slice scanner (Leica, Wetzlar, Germany). Meanwhile, the histological damage scores based on H&E staining were assessed as described previously [25]. Histological scoring was performed in a blinded manner based on the severity of inflammation, epithelial/crypt damage, and enterocyte hyperplasia, with each parameter scored from 0 (normal) to 4 (severe): severity of inflammation (0, normal; 1, mild inflammation; 2, moderate inflammation; 3, obvious inflammation; 4, severe inflammation), extent of epithelial/crypt damage (0, none; 1, less than 10% loss of epithelial structure (crypts); 2, 10–30% crypt loss; 3, 30–50% crypt loss; 4, over 50% crypt loss), and extent of enterocyte hyperplasia (0, normal; 1, focal enterocyte hyperplasia; 2, multifocal enterocyte hyperplasia, and goblet cell loss; 3, diffuse enterocyte hyperplasia, and few goblet cells; 4, diffuse enterocyte hyperplasia and mucosal ulceration).

### 2.6. Inflammatory Cytokines Assay

Determination of interferon-γ (IFN-γ), interleukin 6 (IL-6), interleukin 17F (IL-17F), interleukin 21 (IL-21) and interleukin 10 (IL-10) levels in colonic tissue were performed with ELISA kits (4A Biotech, Beijing, China), and the detection limit for these cytokines were 6 pg/mL, 2 pg/mL, 12 pg/mL, 31 pg/mL and 7 pg/mL, respectively. Colonic tissues were homogenized in 9 volumes of phosphate-buffered saline (PBS, pH 7.0) plus protease inhibitor, and the homogenate was used to analyze cytokines in accordance with the manufacturer’s instructions.

### 2.7. Isolation of Enterocytes and Lymphocytes

After the mouse belly was opened, the colon was quickly excised and placed in cold PBS solution to prevent dehydration. To isolate lamina propria mononuclear cells (LPMCs) and enterocytes, the colon was then longitudinally cut and divided into several pieces (2 cm × 1 cm). The colonic pieces were then rinsed with Hank’s balanced salt solution (HBSS) (gibco) in the presence of 1.5% FBS (gibco) and 1 mM dithiothreitol (Sigma–Aldrich, Darmstadt, Germany) twice and intensively vortexed to deplete feces and mucus. The colonic pieces were then transferred into 5 mL of HBSS containing 1 mM EDTA (Sigma–Aldrich), incubated for 30 min at 37 °C with vigorous shaking at 200 rpm, and then the enterocytes were detached from the lamina propria. Afterward, the remaining colonic lamina propria pieces were incubated with HBSS supplemented with 0.25 mg/mL collagenase type III (Sigma) and 0.2 mg/mL DNase I (Sigma) on an orbital shaker at 100 rpm for 2 h at 37 °C. After digestion, the colonic lamina propria pieces were gently ground to release single cells through the gentleMACS tube, and then the single cells were added onto a 40:80 Percoll (GE Healthcare, Chicago, IL, USA) gradient solution and centrifuged at 2000 rpm for 20 min at room temperature to separate viable LPMCs. The LPMCs were passed through a 70-μm nylon mesh cell strainer and washed twice with complete RPMI medium before use.

### 2.8. Flow Cytometry

Before flow cytometry analysis, the prepared LPMCs or enterocytes were resuspended in staining buffer and incubated with TruStain fcX (BioLegend) to block Fc receptors. For surface marker staining, the cells were stained with antibody solutions for 40 min at 4 °C in the dark. The mAbs labeled with the indicated fluorophore for staining cell surface markers are as follows: anti-CD45 (clone 30-F11, BD Biosciences), anti-CD4 (clone GK1.5, BD Biosciences), anti-CD44 (clone IM7, BD Biosciences), anti-CD62L (clone MEL-14, BD Biosciences), and anti-SLC5A12 (clone H-4, (Santa Cruz Biotechnology, Santa Cruz, CA, USA)). Subsequently, intranuclear transcription factor staining was performed using a fixation/permeabilization kit (BD Biosciences) per the manufacturer’s instructions, and the cells were then stained with antibody solutions for 40 min at 4 °C in the dark. The anti-mouse antibodies used for staining cell intranuclear transcription factor in this study include the following: anti-NRF2 (W19086B, (Proteintech, Rosemont, IL, USA)), anti-Foxp3 (clone 3G3, BD Biosciences), and anti-RORγt (clone Q31-378, BD Biosciences). For intracellular cytokine staining, cells were first stimulated with 0.1 μg/mL phorbol 12-myristate 13-acetate PMA (sigma) and 0.1 μg/mL ionomycin (sigma) in the presence of 5 μg/mL BrefeldinA (1:1000 dilution) at 37 °C for 6 h. After cell-surface staining, the cells were washed, fixed, and permeabilized using fixation/permeabilization buffer solution (BD Biosciences). Next, the fixed cells were stained for intracellular cytokine production using antibody solutions for 40 min at 4 °C in the dark. The following anti-mouse antibodies were used for staining intracellular cytokines: anti-IL-17A (clone TC11-18H10, BD Biosciences), Anti-IFN-γ (clone XMG1.2, BD Biosciences), and Anti-IL-10 (clone JES5-16E3, BioLegend). For the measurement of ROS, enterocytes were stained with 2 mM MitoSOX Red for 10 min at 37 °C in a cell culture incubator. Flow cytometry was detected with a BD LSRFortessa (BD Biosciences), and live cells were discriminated using a Live/Dead Fixable viability dye kit (ThermoFisher, Waltham, MA, USA). Finally, the data were analyzed using FlowJo 10 software, following the gating strategy shown in Figure S1.

### 2.9. Monocytes/T-Cell Coculture

To evaluate the ability of LGG to induce monocyte-mediated polarization of CD4^+^ T cells in vitro, a coculture of PBMCs and naive CD4^+^ T cells was performed. Naive CD4^+^ T cells (CD45^+^ CD4^+^ CD44_low_ CD62L_high_) collected from spleens and lymph nodes of mice were sorted on a BD FACSAria III cell sorter by staining mAbs labeled with the indicated fluorophore. Monocytes collected from the bone marrow of mice were isolated with CD14 microbeads (BioLegend) and were primed with 1 × 10^7^ colony-forming units (CFUs) of LGG lysates that were prepared by pasteurization for 30 min at 70 °C. Naive CD4^+^ T cells were cultured at 150,000 cells/well in the upper chamber of 6-well plates with a Transwell filter membrane (0.4-µm pore polycarbonate membrane insert (Corning, New York, NY, USA)), and 1.5 × 10^6^ monocytes were added to the lower chamber of the Transwell. The cells in the Transwell chamber were incubated in 1.5 mL complete RPMI medium at 37 °C for 24 h. After incubation, CD4^+^ T cells from the upper portion were collected for flow cytometry analysis.

### 2.10. Quantitative Real-Time PCR

For quantification of SLC5A12 mRNA levels, total RNA was extracted from 1 × 10^6^ CD4^+^ T cells using TRIzol reagent (TaKaRa, Dalian, China). Then, the protocols for RNA integrity and quality detection, complementary DNA (cDNA) transcription, and candidate gene q-PCR analysis were performed according to our previous study [26]. Relative expression of SLC5A12 was calculated using the 2^−ΔΔCT^ method [27] with normalization to GAPDH. The SLC5A12-specific primer sequences were forward: TAGGACCCCTGTAGAGGAATATG and reverse: AATGCATCTGTCCACACCACT.

To determine the presence and relative abundance of LGG in fecal pellets, the standard protocol was performed as previously described [21] using LGG-F (5′-CAGCACGTGAAGGTGGGGAC-3′) and LGG-R (5′-CTTGCGGTTGGCTTCAGAT-3′). The genomic DNA of LGG isolated from a pure culture was used for the generation of a standard curve spanning seven points (range: 1 ng µL^−1^–0.015625 ng µL^−1^). The PCR reaction was performed on a CFX96 Real-Time PCR Detection System using a SYBR GREEN PCR Master Mix (TaKaRa), and fecal *L. rhamnosus* GG abundance was normalized by fecal pellet weight.

### 2.11. Biochemical Analysis

For the detection of intracellular lactate, CD4^+^ T cells were isolated from cLP of mice using mouse CD4^+^ T cell microbeads (BioLegend) according to the manufacturer’s instructions. After ultrasonic disruption, intracellular lactate content was determined using a dedicated lactic acid assay kit (Cat# A019-2-2) provided by the Nanjing Jiancheng Bioengineering Institute (Nanjing, China) according to the manufacturer’s instructions.

For the detection of redox metabolites in tissue, colons were isolated from each mouse. After homogenization, the metabolite content of colonic tissue was determined using dedicated quantification kits provided by Nanjing Jiancheng Bioengineering Institute (Nanjing, China) according to the manufacturer’s instructions. The kits for redox metabolite analysis are as follows: Coenzyme I NAD (H) content test kit (Cat# A114-1-1), NADH oxidase (NOX) test kit (Cat# A116-1-1), Hydrogen Peroxide assay kit (Cat# A064-1-1), and Malondialdehyde (MDA) assay kit (Cat# A003-1-2).

### 2.12. Statistical Analysis

Data were analyzed using GraphPad Prism 7. The results are expressed as mean ± standard error of the mean (SEM). Comparisons between two different groups were done by an unpaired two-tailed Student’s *t* test; Comparisons between more than two groups were done by one-way ANOVA (with Tukey’s multiple-comparisons post-tests). A *p*-value < 0.05 was considered significant.

## 3. Results

### 3.1. LGG-Derived Metabolites Induce the Differentiation of Th17 Cells

**Figure 1:**
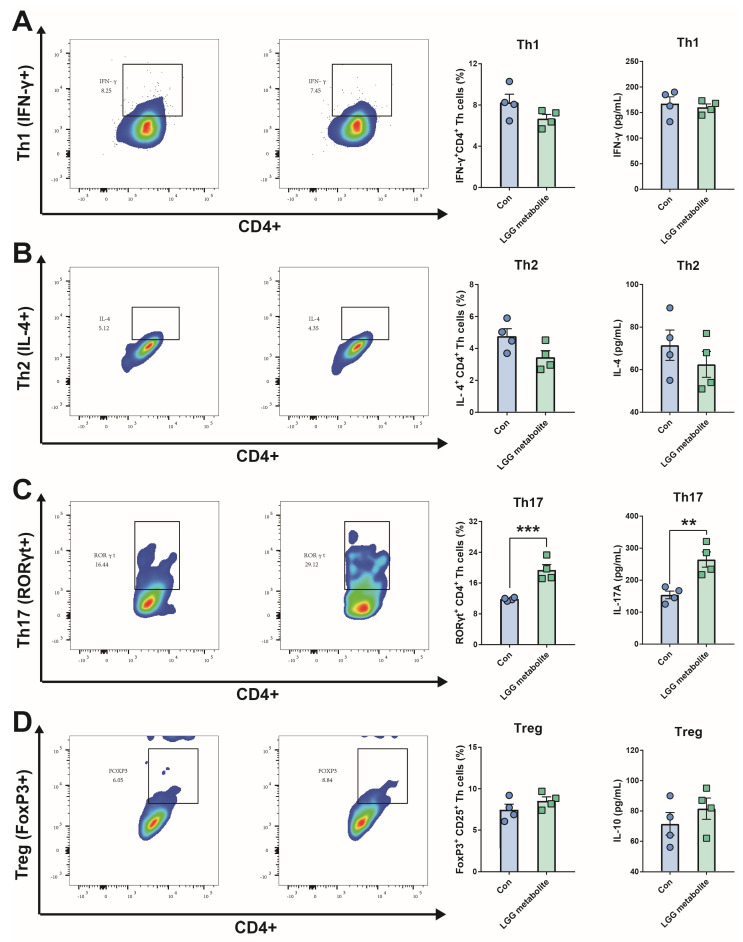
The effects of LGG-derived metabolites on CD4^+^ T cells differentiation in vitro. Naive CD4^+^ T cells isolated from the spleens of C57BL/6 mice were activated with anti-CD3 and anti-CD28 for 24 h. (**A**) Naive CD4^+^ T cells were cultured under Th1 polarization conditions (100 U/mL IL-2; 10 µg/mL anti-IL-4; 10 ng/mL IL-12) for 72 h post-activation. Representative flow cytometry bar graph showing frequencies of IFN-γ^+^ CD45^+^ CD4^+^ T cells in activated CD4^+^ T cells treated with LGG-cell-free supernatant (CFS) or left untreated. Expression of IFN-γ was measured by ELISA kit. (**B**) Representative flow cytometry bar graph showing frequencies of IL-4^+^ CD45^+^ CD4^+^ T cells in activated CD4^+^ T cells treated with LGG CFS or left untreated. Naive CD4^+^ T cells were cultured under Th2 polarization conditions (10 µg/mL anti-IFNγ; 10 ng/mL IL-4) for 72 h post-activation. Expression of IL-4 was measured by ELISA kit. (**C**) Representative flow cytometry bar graph showing frequencies of RORγt^+^ CD45^+^ CD4^+^ T cells in activated CD4^+^ T cells treated with LGG CFS or left untreated. Naive CD4^+^ T cells were cultured under Th17 polarization conditions (50 ng/mL IL-6; 0.5 ng/mL TGF-β1) for 72 h post-activation. Expression of IL-17 was measured by ELISA kit. (**D**) Representative flow cytometry bar graph showing frequencies of Foxp3^+^ CD25^+^ CD4^+^ T cells in activated CD4^+^ T cells treated with LGG CFS or left untreated. Naive CD4^+^ T cells were cultured under Treg polarization conditions (100 U/mL IL-2; 3 ng/mL TGF-β1) for 72 h post-activation. Expression of IL-10 was measured by ELISA kit. Data are shown as mean ± SE. Each dot indicates an individual sample (n = 4). ** *p* < 0.01, *** *p* < 0.001 by one-way ANOVA followed by Tukey’s test.

Recently, evidence suggests that gut commensal microbe-derived metabolites shape the suppressive mucosal immune system, characterized by preferential expansion of Treg cells [28]. Strikingly, *Lactobacillus gallinarum*-derived metabolites mediated the suppression of colonic Treg cells by regulating the IDO1/Kyn/AHR axis [29]; it is tempting to hypothesize that LGG-derived metabolites might influence the differentiation of naive CD4^+^ T cells. To test whether LGG-derived metabolites can induce polarization of CD4^+^ T cells in vitro, splenic naive CD4^+^ T cells were cultured in the presence of soluble αCD3/28 Abs and standardized cytokine combinations with or without metabolites. As presented in Figure 1, although LGG-derived metabolites did not affect the polarization of Tregs, they significantly increased the frequency of RORγt^+^ CD4^+^ T (Th17) cells. In a setting of Th1- or Th2-skewing conditions, we also observed that LGG-derived metabolites had no effects on the differentiation of Th1 or Th2. Compared with control naive CD4^+^ T cells, naive CD4^+^ T cells cultured with LGG metabolites showed a 1.5-fold increase in IL-17 secretion, but there was no obvious increase in IFN-γ, IL-4, or IL-10.

### 3.2. LGG-Derived Metabolites Modulate SLC5A12 Transporter in CD4^+^ T Cells

**Figure 2:**
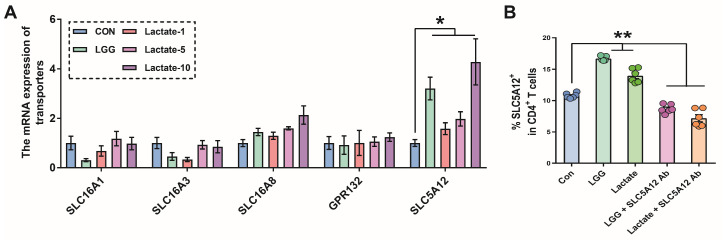
LGG-derived lactate activates SLC5A12 in naive CD4^+^ T cells. (**A**) qPCR analysis of the indicated transporter genes in naive CD4^+^ T cells treated with LGG CFS, or different amounts of Lactate (1 mM, 5 mM, 10 mM), or left untreated (n = 4). (**B**) Representative flow cytometry bar graph showing frequencies of SLC5A12^+^ CD4^+^ T cells in activated CD4^+^ T cells treated with Lactate (10 mM) with or without SLC5A12 Ab, or *A. muciniphila* CFS with or without SLC5A12 Ab, or left untreated (n = 6). Data are shown as mean ± SE. Each dot indicates an individual sample. * *p* < 0.05, ** *p* < 0.01 by one-way ANOVA followed by Tukey test.

The *lactobacillus* species produce large amounts of lactate, which has recently been suggested to possess pro-inflammatory properties promoting Th17 cell polarization [21]. Several studies have sought a cross-relationship between lactobacillus-derived lactate and the transporters (i.e., SLC16A1, SLC16A3, and SLC5A12) for lactate [20]. Here, we interrogated which transporters of CD4^+^ T cells respond to lactobacillus-derived lactate by q-PCR. After αCD3/28 Abs activation, we observed that the expression of *SLC5A12* was upregulated by lactobacillus-derived metabolites, whereas the expression of other transporters, including *SLC16A1*, *SLC16A3*, *SLC16A8*, and *GPR132*, was not changed. To further determine the direct role of lactate in transporters, the expression of related membrane transporters for lactate was measured following activation of CD4^+^ T cells exposed to lactate. Similarly, lactate selectively promoted the expression of *SLC5A12* in naive CD4^+^ T cells, which exhibited dose-dependent effects (Figure 2A). We used flow cytometry analysis to further confirm that increased *SLC5A12* expression in CD4^+^ T cells occurred when cells were exposed to lactate, but not in the presence of SLC5A12 Ab, which inhibited the transport of lactate (Figure 2B). These findings suggest that LGG-derived lactate is mainly transported into CD4^+^ T cells through the SLC5A12 transporter.

### 3.3. SLC5A12 Ab Reinforces the Therapeutic Effects of Oral Live LGG on Colitis in the Mouse Model

**Figure 3:**
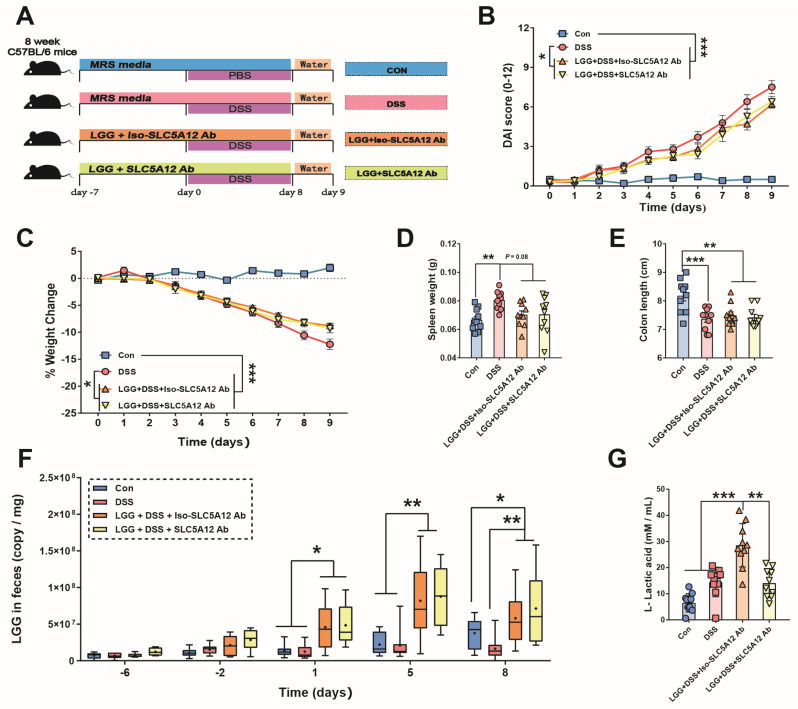
LGG alleviates colitis in mice induced by DSS. To evaluate the therapeutic effects of LGG against inflammatory colitis, we used a well-established acute colitis model where the disease is induced by DSS. We administered oral gavage (daily) to mice with live LGG starting 1 week before DSS treatment, and on the third day post-DSS treatment, mice were treated by intraperitoneal injection of anti-SLC5A12 or its isotype antibody. (**A**) Experimental design of SLC5A12 Ab injection in SPF C57BL/6 mice, orally receiving control medium or LGG starting 1 week before 2.5% DSS treatment. Mice were treated by intraperitoneal injection of anti-SLC5A12 or its isotype antibody on day 3 following DSS treatment. On the eighth day post-DSS treatment, all mice were given regular water for 24 h and followed by sacrifice (n = 10). (**B**–**E**) Disease activity index (DAI) (**B**), weight changes (**C**), spleen weight (**D**), and colonic length (**E**) of SPF C57BL/6 mice post-DSS treatment. (**F**) The abundance of LGG in fecal samples of indicated mice at different time points post-DSS treatment. (**G**) The concentration of lactic acid from CD4^+^ T cells in cLP isolated from the indicated SPF mice. Data are shown as mean ± SE. Each dot indicates an individual sample. * *p* < 0.05, ** *p* < 0.01, *** *p* < 0.001 by two-way ANOVA followed by Bonferroni post hoc test (**B**,**C**) or one-way ANOVA followed by Tukey test (**D**,**E**,**G**).

To evaluate the therapeutic effects of LGG against inflammatory colitis, we used a well-established acute colitis model [30] where the disease is induced by DSS. We administered oral gavage (daily) to mice with live LGG starting one week before DSS treatment, and on the third day post-DSS treatment, mice were treated by intraperitoneal injection of anti-SLC5A12 or its isotype antibody (Figure 3A). We observed that oral gavage with live LGG alleviated DSS-induced weight loss (Figure 3C), decreased the disease activity index (Figure 3B) and spleen weight (Figure 3D), as well as the shortening of colon length (Figure 3E). Next, we performed q-PCR on fecal samples with LGG-specific primers, and this assay concluded that LGG abundance was elevated in the colonic luminal in DSS-treated mice administered with LGG in combination with the SLC5A12’s isotype control antibody (LGG + Iso-SLC5A12 Ab mice), and the LGG abundance in the colonic lumen of DSS-treated mice administered with LGG in combination with the SLC5A12 antibody (LGG + SLC5A12 Ab mice) increased to similar degrees (Figure 3F). However, SLC5A12 blockade resulted in a lower intracellular lactate concentration in CD4^+^ T cells isolated from cLP than treatment with the isotype control antibody (Figure 3G).

**Figure 4:**
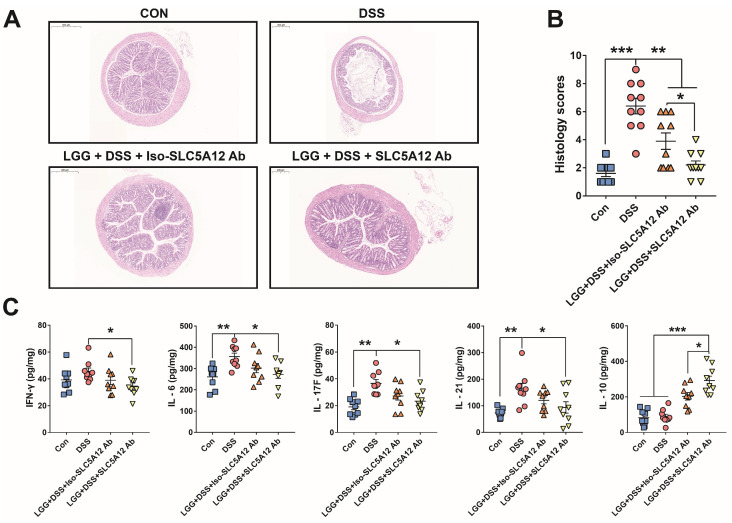
LGG mitigates the histopathological colon injury and inflammatory response. (**A**,**B**) Representative colonic histological images (Scale bars, 500 μm) (**A**) and colonic histological score (**B**) of mice post-DSS treatment. (**C**) The secretion of cytokine isolated from the colon of C57BL/6 mice was assessed by ELISA. Data are shown as mean ± SE. Each dot indicates an individual sample. * *p* < 0.05, ** *p* < 0.01, *** *p* < 0.001 by two-way ANOVA followed by Bonferroni post hoc test (**B**,**C**) or one-way ANOVA followed by Tukey’s test.

Strikingly, although oral LGG administration improved DSS-worsening mucositis as indicated by the decreased histological inflammatory scoring, SLC5A12 Ab further reduced the score compared to its isotype control antibody (Figure 4A,B). We next assessed expression levels of some pro-inflammatory cytokines (e.g., IFN-γ, IL-6, IL-17F, and IL-21) and anti-inflammatory cytokines, such as IL-10; these inflammatory cytokines participate in the inflammatory colitis process. The whole large intestinal fraction isolated from DSS-treated only mice produced significantly more IL-6, IL-17F, and IL-21 than tissue cytokine levels isolated from non-colitic control mice or LGG + SLC5A12 Ab mice (Figure 4C). Nevertheless, no difference in pro-inflammatory cytokine levels was found between the LGG + Iso-SLC5A12 Ab mice and DSS-treated only mice (Figure 4C). Of note, oral LGG administration elevated the level of IL-10 in the large intestine under inflammatory conditions, and SLC5A12 Ab further increased the content of IL-10 compared to treatment with the isotype control antibody in vivo (Figure 4C).

### 3.4. LGG Improves Intestinal Th17/Treg Homeostasis Under the SLC5A12 Transporter Inhibition Conditions

**Figure 5:**
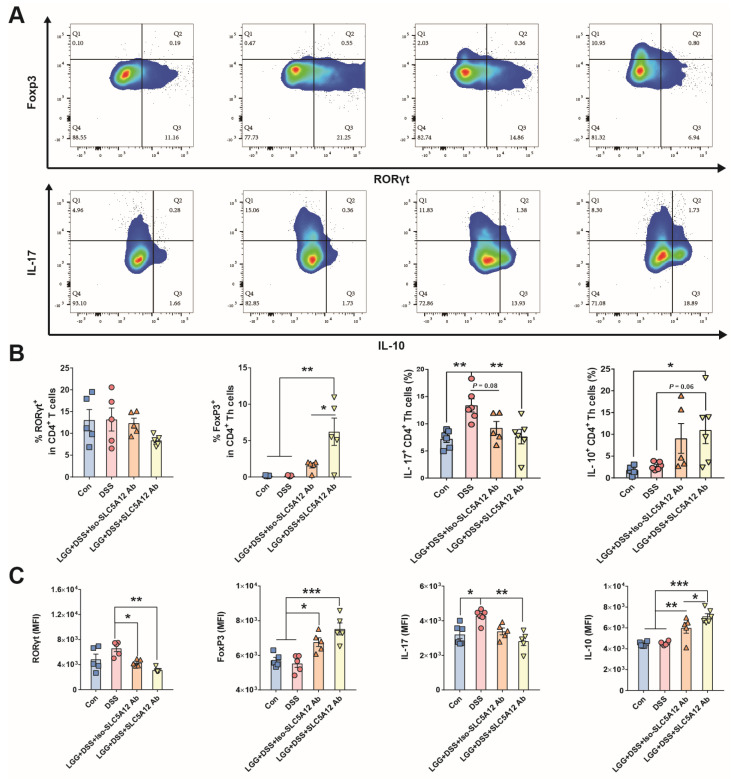
Oral administration of LGG inhibited autoimmunity by modulating Th17/Treg balance. (**A**–**C**) Representative flow cytometry plots (**A**) and bar graphs (**B**) showing frequencies and mean fluorescence intensity (MFI) (**C**) of RORγt^+^ CD4^+^ T cells, Foxp3^+^ CD4^+^ T cells, IL-17^+^ CD4^+^ T cells, and IFN-γ^+^ CD4^+^ T cells in intestinal lamina propria of indicated mice. Data are shown as mean ± SE. Each dot indicates an individual sample. * *p* < 0.05, ** *p* < 0.01, *** *p* < 0.001 by one-way ANOVA followed by Tukey’s test.

To better understand how oral LGG administration activates inflammatory immune responses, we performed flow cytometry analysis of cLP isolated from mice (Figure 5A). Similar to the results of the inflammatory cytokines analysis in intestinal tissues, DSS-treated only mice displayed an increased IL-17^+^ cell frequency (Figure 5B) and mean fluorescence intensity level (Figure 5C). Compared with DSS-treated only mice, decreased Th17 cell differentiation was found in cLP of LGG + SLC5A12 Ab mice. Conversely, we observed that in cLP of LGG + SLC5A12 Ab mice, the percentage of Foxp3^+^ cells significantly increased, which is precisely the characteristic of Treg cells. Although the percentage of Foxp3^+^ cells was also increased in cLP of LGG + Iso-SLC5A12 Ab mice, SLC5A12 Ab resulted in a higher percentage of Foxp3^+^ cells than the isotype control antibody. We also consistently observed an increase in the percentage of IL-10^+^ cells in cLP of LGG + SLC5A12 Ab mice.

### 3.5. In Vitro Priming of Naive CD4^+^ T Cells with LGG Induces Treg Cells

**Figure 6:**
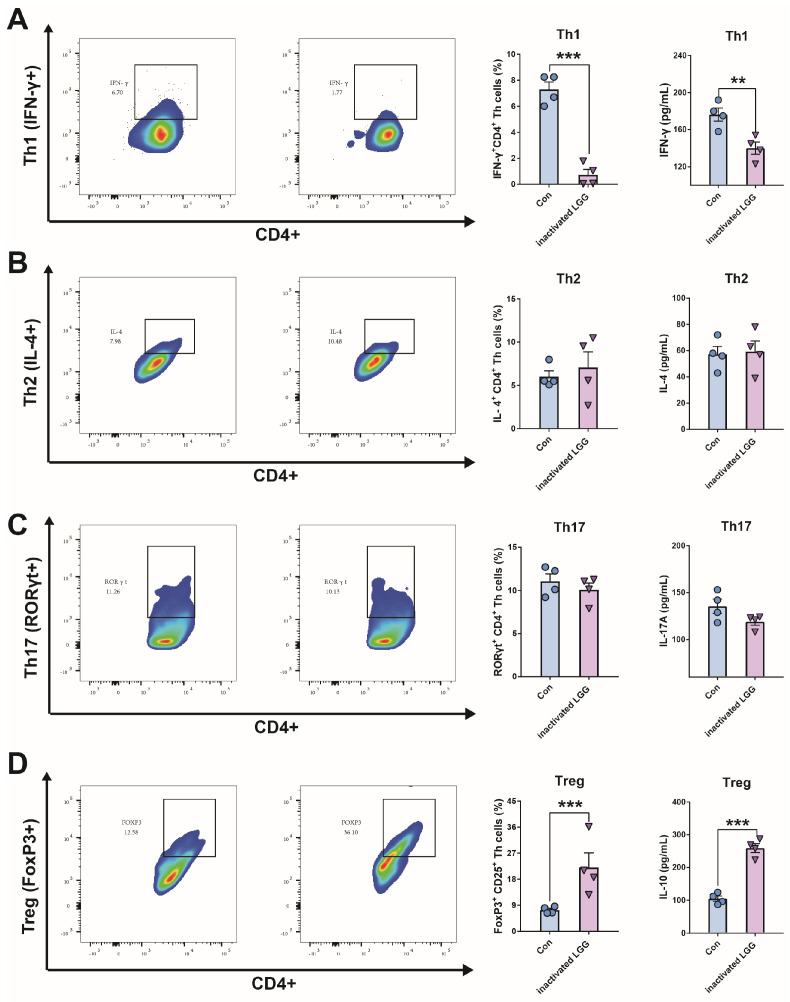
CD4^+^ T cells primed with heated LGG induce Treg-polarizing cells. Naive CD4^+^ T cells isolated from spleens of C57BL/6 mice were co-cultured with autologous monocytes pulsed with heat-inactivated LGG, for 3 days. After the polarization was completed, cells were stained with transcription factor antibodies to ROR-γt for determining Th17 (**C**) or Foxp3 for determining Treg (**D**), or stimulated for 6 h with PMA and ionomycin, fixed, permeabilized, and stained with cytokine antibodies to IFN-γ for determining Th1 (**A**) or IL-4 for determining Th2 (**B**). Expression of IFN-γ, IL-4, IL-17A and IL-10 was measured by ELISA kit (**A**–**D**). Data are shown as mean ± SE. Each dot indicates an individual sample (n = 4). ** *p* < 0.01, *** *p* < 0.001 by one-way ANOVA followed by Tukey’s test.

It has been reported that *Lactobacillus reuteri* elicited the intestinal autoreactive CD4^+^ T cell response through specific antigen peptides [31]. We explored whether CD4^+^ T cell differentiation initiated by antigen presentation can be reprogrammed by probiotic LGG. Here, we adopted an antigen-specific CD4^+^ T cell-priming approach [32] using autologous monocytes pulsed with heat-inactivated LGG as antigen-presenting cells. When naive CD4^+^ T cells were cultured with monocytes pulsed with inactivated LGG, antigen-specific proliferating T cells could be detected after three days. As shown in Figure 6, in comparison to non-primed monocytes, LGG-primed monocytes enabled CD4^+^ T cells to produce similar levels of cytokine IL-4 and the transcription factor RORγt, but did not produce IFN-γ. Importantly, LGG-primed monocytes specifically resulted in a substantial fraction of CD4^+^ T cells acquiring the capacity to express Foxp3. These findings indicate that specific T cell responses to LGG-primed monocytes can be generated in vitro, leading to Treg cell polarization. Compared with control CD4^+^ T cells, CD4^+^ T cells cultured with LGG-primed monocytes showed a 1.5-fold increase in IL-10 secretion and a dramatic decrease in IFN-γ secretion. However, the production of IL-17A and IL-4 was not changed.

### 3.6. LGG Scavenges Intracellular ROS Through Inducing Nrf2 Expression in Enterocytes

**Figure 7:**
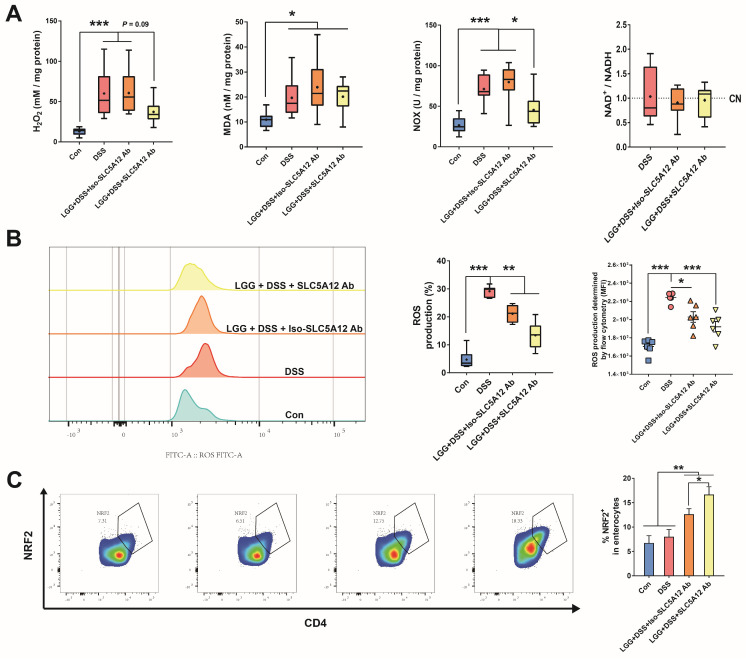
LGG scavenges intracellular ROS through reinforcing Nrf2 expression in colonic enterocytes. Experimental design of SLC5A12 Ab injection in SPF C57BL/6 mice, orally receiving control medium or LGG. At day 0, 2.5% DSS or normal drinking water was given for 8 days, followed by regular water for 1 day. Mice were sacrificed at day 9 after DSS treatment (n = 10). (**A**) H_2_O_2_, MDA, NOX, NAD^+^, and NADH levels in colonic tissue isolated from indicated mice (n = 10). (**B**) Representative flow cytometry peak graphs and bar graphs showing frequency and mean fluorescence intensity (MFI) of ROS in colonic enterocytes of indicated mice (n = 6). (**C**) Representative flow cytometry plots and bar graph showing frequency of Nrf2^+^ enterocytes isolated from the colon of indicated mice (n = 3). Data are shown as mean ± SE. Each dot indicates an individual sample. * *p* < 0.05, ** *p* < 0.01, *** *p* < 0.001 by two-way ANOVA followed by Bonferroni post hoc test (**A**) or one-way ANOVA followed by Tukey’s test (**B**,**C**).

Recently, studies have shown that ROS contribute to the development of autoimmunity, which is essential for shaping the intestinal pathology phenotype. Based on these works, we investigated whether LGG plays a pivotal role in removing ROS. We observed that the amount of hydrogen peroxide (H_2_O_2_) and NOX, which reflect mitochondrial respiration, were significantly lower in colonic tissues from LGG + SLC5A12 Ab mice (Figure 7A). Then, we performed flow cytometry analysis of ROS generation in colonic enterocytes, and found that the frequency and mean fluorescence intensity of ROS cells were decreased in enterocytes of DSS-treated mice administered with LGG. We also found that SLC5A12 Ab reduced ROS levels in colonic enterocytes to a certain extent compared to its isotype control antibody (Figure 7B). Of particular note, Nrf2, an antioxidant nuclear factor, was increased in colonic enterocytes from DSS-treated mice administered with LGG. In addition, SLC5A12 blockade further results in an increase in the percentage of Nrf2^+^ enterocytes than treatment with the isotype control antibody (Figure 7C).

## 4. Discussion

Preclinical studies using probiotics, such as LGG, have clearly shown promise for patients with antibiotic-associated diarrhea [10], Helicobacter pylori-related dyspepsia [11], infant colic [12], and other gastrointestinal indications [13]. In recent years, accumulating evidence has demonstrated that LGG appears to regulate the Th17/Treg balance [16,18,19], which might develop into a potential treatment for IBD. It is worth noting that the results of research about LGG’s impact on the Th17/Treg balance are contradictory: on the one hand, increased levels of Treg-associated cytokines, including IL-10 and TGF-β, were observed in orally LGG-treated mice [19]; on the other hand, there is a growing trend of studies finding that LGG shows a potential ability to increase Th17 cells via its specific epitope for TCR [18] of naïve CD4^+^ cells or its metabolite secretions–lactate [20]. Based on these works, we employed two types of priming CD4^+^ cell models and the DSS-induced acute murine colitis model, which illustrated the underlying mechanisms by which LGG exerted immunomodulatory properties and improved the IBD pathology phenotype. In particular, we identified SLC5A12, a lactate transporter, as playing a vital role in shaping the Th17/Treg balance through directing the differentiation of naive CD4 cells.

We first demonstrated that the metabolite of LGG significantly augmented Th17 cell generation and induced remarkable Rorγt^+^ expression in vitro. Several studies suggested that host–commensal metabolic interactions lead to immunity signaling activation that, in turn, promotes inflammatory response-related intestinal pathologies. For instance, Iatsenko et al. demonstrated that *Lactobacillus plantarum* promotes intestinal damage through the release of lactate in the fly gut [33]. Although lactate has long been considered a waste product of aerobic glycolysis, recent findings have identified it as a signal molecule that modulates effector function and differentiation in CD4^+^ cells [20]. In a murine model of psoriasis, lactate derived from epithelial cells robustly enhanced the type 17 γδ T cell response in inflamed skin [34]. Strikingly, the increased lactate accumulation in the intestinal cavity of patients with IBD is accompanied by the excessive activation of mucosal Th17 lymphocytes [35]. Correspondingly, the transport of lactate is dependent on various transporters, including sodium-dependent transporters (i.e., SLC5A8 and SLC5A12) or proton-lactate symport transporters (i.e., SLC16A1 and SLC16A3) [20]. Indeed, we found that only SLC5A12, which is selectively expressed in CD4^+^ cells, exhibited a high affinity for lactate, consistent with the results of a previous study [21]. Based on the evidence of increased expression levels of SLC5A12 on the surface of naïve CD4^+^ cells, we postulate that the metabolites of LGG modulate the Th17 cell response via SLC5A12.

We then adapted a combination treatment of LGG and SLC5A12 Ab to colitis mice to explore the therapeutic potential of LGG for IBD from several perspectives. Although the DSS model cannot fully replicate the complex heterogeneity of human inflammatory bowel disease, given its reversible disease course and non-genetic induction, it remains a powerful and widely used tool for studying intestinal inflammation and screening potential therapeutics. Meanwhile, to minimize experimental variability caused by hormonal fluctuations, this study exclusively used male mice to ensure more stable and reliable results. Consistent with previous literature [36], our research findings indicated that the DSS challenge increased the DAI score, weight loss, spleen index, and colon atrophy, which were mitigated by LGG. On top of this, oral administration of LGG was associated with increased LGG distribution in the intestinal tract and lactate accumulation within CD4^+^ cells, indicating the excellent colonization ability of LGG. Intestinal morphology is usually presented through HE staining, which is an important indicator of epithelial/crypt damage, inflammation, or even enterocyte hyperplasia [25]. Previous studies have confirmed that the anti-SLC5A12 monoclonal antibody shows a significant inhibitory effect on the transport of lactate into and out of T cells. Moreover, SLC5A12 Ab reduced and even blocked the pro-inflammatory and pathogenic effects of lactate by targeting transporters in arthritic tissues [20]. In this study, while LGG supplementation decreased histology scores in the colon of mice treated with DSS, SLC5A12 Ab treatment further promoted the anti-colitic effects of LGG. This may be due to the role of SLC5A12 for the transportation of lactate, which is reported to influence the Th17 response. All in all, other outcomes, such as immunomodulatory effects of LGG in vivo, were explored to clearly elucidate the changes in intestinal morphology.

Previous studies have highlighted the role of the Th17/Treg balance in maintaining normal intestinal homeostasis [2,3]. Hence, we firmly believe that LGG could alleviate the inflammatory response in the DSS-induced colitis murine model via its capability to modulate the differentiation of naïve CD4^+^ cells. Similar to recent findings in rats [37], our results confirmed that treatment with LGG is sufficient to increase CD45^+^ CD4^+^ Foxp3^+^ Treg cells within the lamina propria. In addition, additional treatment with the lactate transporter monoclonal antibody, SLC5A12 Ab, in vivo leads to a decrease in Rorγt^+^ Th17 cells, which in turn may be more conducive to the differentiation of Treg cells. Consistently, we observed that cotreatment with LGG and SLC5A12 Ab not only decreased the levels of Th17-characteristic cytokines such as IL-17 and IL-21, as well as the Th17 cell–polarizing cytokine IL-6, but also increased the level of the anti-inflammatory cytokine IL-10 in colonic tissue. Nonetheless, the current knowledge regarding the role of LGG in regulating the response of Treg cells by modulating TCR signals is extremely limited. Another study indicated that LGG intervention influences the balance between Th17 and Treg, which is accompanied by the enrichment of the TCR signaling pathway determined via KEGG pathway analysis [38]. Here, we hypothesize that LGG modulates Treg cell development in a metabolite-independent manner.

To verify the hypothesis that LGG might boost Treg cells in naïve CD4^+^ cells by priming TCR signaling via a specific antigen, we employed a cell co-culture method in which antigen-primed monocytes were mixed with and cultured with naïve CD4^+^ cells. Here, we observed that LGG-loaded autologous monocytes enabled naïve CD4^+^ cells to acquire the ability to express Foxp3, which is characteristic of Treg cells. Conversely, it has been reported that an invariant Vγ6Vδ1 TCR specific for an epitope from LGG, which corresponds to IL17-committed γδ T cells of the lung in newborn mice [18]. As a matter of fact, upon the stimulation of specific microbial antigens, the development of different CD4^+^ T cell subsets is largely dependent on antigen-presenting cells (e.g., dendritic cells (DC), macrophages, and B lymphocytes) [39]. In healthy mice, *L. plantarum* was reported to elicit expansion of regulatory CD103^+^ DCs, thereby increasing the frequency of Treg cells [40]. A study indicated that LGG can alleviate colonic inflammation by forming an IL-10-based autocrine loop in Ly6C^+^ monocytes [41]. Potentially, the elevated Treg frequency can be explained by the role of LGG in APCs, and future research is required to address the underlying mechanism behind this. Notably, in contrast to the well-documented anti-inflammatory effects of the probiotic LGG, its derived metabolites were found to promote Th17 differentiation in vitro. Previous studies found that lactate might promote Th17 cell differentiation through reactive oxygen species (ROS)-mediated activation of transforming growth factor-beta (TGF-beta) [42,43]. Although TGF-beta and IL-6 together are reported to induce the generation of Th17 cells from naive T cells, TGF-beta is also a key factor for the differentiation of Treg cells. Moreover, IL-6 can completely inhibit the differentiation of Treg cells induced by TGF-beta [44]. Interestingly, LGG-derived exopolysaccharides (EPSs) can decrease the content of the pro-inflammatory cytokine IL-6 in the brain tissues of D-galactose-treated mice [45]. Thus, we speculate that LGG can inhibit the key pro-inflammatory factor IL-6 through anti-inflammatory EPSs, thereby masking the pro-Th17 generation signal mediated by its metabolite lactate. Although our data indicate that LGG exerts a dual effect, eliciting both pro-inflammatory and regulatory signals, the precise mechanisms by which it configures these conflicting signals under specific conditions—particularly in enteritis—and how it prioritizes the activation of anti-inflammatory pathways remain to be further elucidated.

It has long been proposed that ROS, triggered by the catalytic action of NADPH oxidases (e.g., Duox and Nox), are responsible for the association between chronic inflammatory response and elevated IBD incidence [46]. A previous study [33] had indicated that ROS lead to intestinal damage by inducing intestinal dysplasia, manifested as excessive proliferation of intestinal stem cells. Furthermore, current reports also found that ROS pathways contributed to the enhanced Th17 cell differentiation in the gut mucosa [47]. Intriguingly, although LGG is deficient in both the superoxide dismutase (SOD) gene and catalase (CAT) gene in the chromosome [48], it can still effectively inhibit ROS overproduction within gastrointestinal epithelial cells [6]. In line with this, we confirmed that the LGG reduced levels of ROS, H_2_O_2_, and MDA, as well as the enzymatic activity of NOX within colonic enterocytes. Additionally, the mean fluorescence intensity of ROS enterocytes was further decreased upon SLC5A12 Ab injection, which might be because lactate acts as a substrate in the tricarboxylic acid cycle that triggers ROS production [49]. Mechanistically, our results provided evidence that LGG upregulated the expression of intracellular Nrf2, which combines with the nuclear antioxidant response element sequence, thereby inducing antioxidant enzymes (e.g., SOD and Glutathione peroxidase (GSH-Px) transcription, which is similar to the results of a previous study [50]).

## 5. Conclusions

Therefore, our data suggest that dietary LGG can serve as an effective food additive that can beneficially improve symptoms of preclinical colitis by modulating Th17/Treg balance, and SLC5A12 blockade appears to enhance the anti-inflammatory properties of LGG, thereby providing a theoretical basis for developing a nutritional adjunct-to-standard therapy strategy for improving colitis.

## Data Availability

The data supporting the conclusions of this article are included within the article. Additional data used and/or analyzed during the current study are available from the corresponding authors upon reasonable request.

## Supplementary Materials

The following supporting information can be downloaded at: https://www.mdpi.com/article/10.3390/nu18111724/s1, Figure S1: The figure shows gating strategy of flow cytometry.

## Abbreviations

The following abbreviations are used in this manuscript:

LGG	*Lactobacillus rhamnosus* GG
DSS	dextran sulfate sodium
SLC5A12	lactate-specific transporter solute carrier family 5 member 12
Nrf2	nuclear factor-erythroid 2-related factor 2
IBD	inflammatory bowel disease
TLR2	toll-like receptor 2
cLP	colonic lamina propria
TCR	T cell receptor
SCFAs	short-chain fatty acids
H&E	Hematoxylin and Eosin
IFN-γ	interferon-γ
IL-6	interleukin 6
LPMCs	lamina propria mononuclear cells
HBSS	Hank’s Balanced Salt Solution
CFUs	colony-forming units
cDNA	complementary DNA
NOX	NADH oxidase
MDA	malondialdehyde
H_2_O_2_	hydrogen peroxide
SOD	superoxide dismutase
CAT	catalase
GSH-Px	glutathione peroxidase
