# Supplementary material for: Lactobacillus rhamnosus GG Alleviates Colitis by SLC5A12-Mediated Th17/Treg Cell Balance in Mice
Source: Nutrients. 2026 May 28;18(11):1724. doi: 10.3390/nu18111724 (PMC13258439; doi:10.3390/nu18111724)
Supplement: Supplementary file 1 [file nutrients-18-01724-s001.zip › nutrients-4287567-supplementary.pdf]

Supplementary File

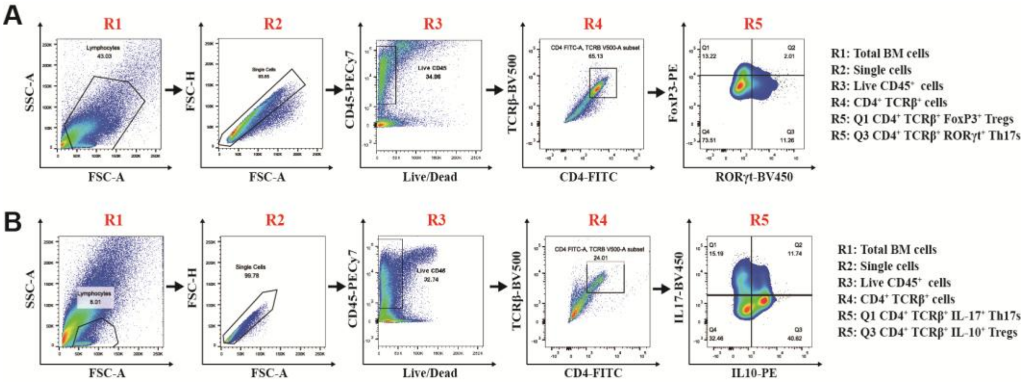

Supplementary Figure S1. The figure shows gating strategy of flow cytometry.

(A) Gating strategy used to identify RORγt^{+} CD4^{+} TCRβ^{+} cells or Foxp3^{+} CD4^{+} TCRβ^{+} cells by flow cytometry.

(B) Gating strategy used to identify IL-17A^{+} CD4^{+} TCRβ^{+} cells or IL-10^{+} CD4^{+} TCRβ^{+} cells by flow cytometry.

Following being washed with PBS, single cell suspensions were stained with antibodies to the indicated antigens and live/dead cell dye. Gated regions are numbered from R1 to R5. The figure shows one representative gating of flow cytometric plot.
